# Transcriptomic Analysis Reveals an Altered Hcy Metabolism in the Stria Vascularis of the Pendred Syndrome Mouse Model

**DOI:** 10.1155/2021/5585394

**Published:** 2021-04-17

**Authors:** Wenyue Xue, Yuxin Tian, Yuanping Xiong, Feng Liu, Yanmei Feng, Zhengnong Chen, Dongzhen Yu, Shankai Yin

**Affiliations:** ^1^Department of Otolaryngology-Head and Neck Surgery, Shanghai Jiao Tong University Affiliated Sixth People's Hospital, Shanghai, China; ^2^Otolaryngology Institute of Shanghai Jiao Tong University, Shanghai, China; ^3^Shanghai Key Laboratory of Sleep Disordered Breathing, Shanghai, China; ^4^Department of Otolaryngology-Head and Neck Surgery, First Affiliated Hospital of Nanchang University, Nanchang, Jiangxi, China

## Abstract

**Purpose:**

*Slc26a4^−/−^* mice exhibit severer defects in the development of the cochlea and develop deafness, while the underlying mechanisms responsible for these effects remain unclear. Our study was to investigate the potential mechanism linking *SLC26A4* deficiency to hearing loss.

**Materials and Methods:**

RNA sequencing was applied to analyze the differential gene expression of the stria vascularis (SV) from wildtype and *Slc26a4^−/−^* mice. GO and KEGG pathway analysis were performed. Quantitative RT-PCR was applied to validate the expression of candidate genes affected by *Slc26a4*. ELISA and immunofluorescence technique were used to detect the homocysteine (Hcy) level in serum, brain, and SV, respectively.

**Results:**

183 upregulated genes and 63 downregulated genes were identified in the SV associated with *Slc26a4* depletion. Transcriptomic profiling revealed that *Slc26a4* deficiency significantly affected the expression of genes associated with cell adhesion, transmembrane transport, and the biogenesis of multicellular organisms. The SV from *Slc26a4^−/−^* mice exhibited a higher expression of *Bhmt* mRNAs, as well as altered homocysteine (Hcy) metabolism.

**Conclusions:**

The altered expression of *Bhmt* results in a dramatic change in multiple biochemical reactions and a disruption of nutrient homeostasis in the endolymph which may contribute to hearing loss of *Slc26a4* knockout mouse.

## 1. Introduction

Pendred syndrome, characterized by deafness with enlargement of aqueduct and goiter, is caused by mutations of *SLC26A4*, one of the most prevalent causes of hereditary hearing loss globally [1]. A large-scale study showed mutations of *SLC26A4* that occur in approximately 5-10% sensorineural hearing loss children among a variety of different ethnic populations [2]. The onset of hearing loss in patients with Pendred syndrome is variable. Some patients may have hearing loss at birth, while others suffer fluctuating, or progressive hearing loss during their childhood, which may provide a window of opportunity for treatment [3].

To investigate the underlying mechanism of the Pendred syndrome, researchers have created a mouse model for study. The *Slc26a4* knockout mice suffer severe hearing loss and malformation of cochlea at birth. Previous studies looking at the *Slc26a4* knockout mice have found that pendrin, encoded by the *Slc26a4* gene, locates in root cells of the outer sulcus, cells overlying the spiral prominence, and spindle-shaped cells of the stria vascularis in cochlea. Meanwhile, three main changes occur in the inner ears of *Slc26a4* mutant mouse. (1) A lack of pendrin causes the acidification of the endolymph [4]. Pendrin is an anion exchange protein, similar to Cl^−^ and HCO_3-_ exchangers that participate in fluid regulation of the endolymph [5]. In the absence of pendrin, HCO_3-_ secretion into the endolymph is affected, which causes the endolymph to become acidic and leads to inhibition of Ca^2+^ reabsorption [4, 6]. (2) The scala media is enlarged due to the dysfunction of the endolymphatic sac. The cochlear lumen is formed by the balance of the fluid secretion in the vestibular labyrinth and fluid absorption in the endolymphatic sac, from embryonic day (E) 13.5 and 14.5 [7]. Thus, dysfunction of fluid absorption in the endolymphatic sac, due to *Slc26a4* mutation, results in an enlarged volume of the scala media [8, 9]. (3) The expression of Kcnj10 is reduced, and this inwardly rectifying K*^+^* channel subunit, located in the intermediate cells of the stria vascularis (SV), is responsible for the establishment of the endolymphatic potential (EP) [10, 11]. Wangemann et al. have reported an absence of Kcnj10 in the intermediate cells of *Slc26a4* mutant mice at 1-4 months of age, and this lack of channel protein causes a decrease in the EP, which may directly lead to deafness [12]. Among these three changes, the pathology in the SV is the initial and predominant cause of the hearing loss associated with *Slc26a4^−/−^* mice. However, how *Slc26a4* deficiency affects morphology and function of SV remains unknown.

In this study, we used RNA sequencing methodology to compare differences in the transcriptomes in the SV from wildtype and *Slc26a4* mutant mice. We found that *Slc26a4* deficiency significantly affected SV genes required for multicellular organism biogenesis. Furthermore, the SV from *Slc26a4^−/−^* mice exhibited higher expression of *Bhmt* mRNAs, as well as a dramatic reduction of Hcy, suggesting abnormality of Hcy metabolism as a novel mechanism by which *SLC26A4* affects hearing.

## 2. Materials and Methods

### 2.1. Animals

*Slc26a4* knockout mice were obtained from the Jackson Laboratory, and *Calca* knockout mice were from Cyagen Co. LTD. All animals were maintained in heterozygotes in SPF circumstances and adapted to the experimental circumstances for a week before the experiments. All experimental procedures were carried out in accordance with the Guide for the Care and Use of Laboratory Animals and were approved by the Animal Care and Use Committee of the Shanghai Jiao Tong University Affiliated Sixth People's Hospital [Permit number: SYXK 2016-0020].

### 2.2. RNA-seq

Wildtype and *Slc26a4^−/−^* mice were sacrificed at P14. The stria vascularis were isolated from inner ear and then lysed in Trizol (Invitrogen). Total RNA was isolated according to the manufacturer's instructions. All samples were sequenced on an Illumina HiSeq2500 platform at 15 million 100-bp single reads per sample. After quality control of the sequencing libraries, reads were trimmed and mapped against the Ensembl genome annotation and the human genome assembly (hg19/GRCh38) using Tophat2. Reads mapping to ribosomal RNAs or the mitochondrial genome were removed.

### 2.3. Quantitative Real-Time RT-PCR

Total RNA from wildtype and *Slc26a4^−/−^* stria vascularis was extracted with TRIzol reagent according to the manufacturer's instructions (Invitrogen). One microgram of total RNA was reverse transcribed using the ReverTra Ace® qPCR RT Kit (Toyobo, FSQ-101) according to the manufacturer's instructions. A SYBR RT-PCR kit (Toyobo, QPK-212) was used for quantitative real-time PCR analysis. The relative mRNA expression of different genes was calculated by comparison with the control gene *Gapdh* (encoding GAPDH) using the 2^-*△△*Ct^ method (Table 1).

### 2.4. Immunostaining

Wildtype and *Slc26a4^−/−^* mice were sacrificed at P14. The cochleae were isolated from temporal bone, fixed by 4% PFA, dehydrated by 30% sucrose, and cut into frozen sections. The section was then permeabilized with 0.1% Triton X-100 in PBS and blocked with 1% BSA in PBS. The samples were incubated with anti-Hcy (PAD984Ge01, Clonecloud) at 4°C overnight and then secondary antibody or DAPI at room temperature for 30 min. The stria vascularis were dissected from lateral wall and its actin structure were shown by phalloidin staining. Samples were examined and the figures were acquired with an LSM 710 confocal laser-scanning microscope (Carl Zeiss, Oberkochen, Germany) at 20x or 63x magnification.

### 2.5. Enzyme-Linked Immunosorbent Assay

The blood plasma and brain homogenate from wildtype and *Slc26a4^−/−^* mice were prepared and persevered at -20°C until analysis. Hcy level was measured using ELISA Kit for Homocysteine (CED984Ge, Clone-Cloud) according to the manufacture's instruction. Absorbance was assessed by an ELISA microplate reader at 450 nm and the results were expressed in pg/ml.

### 2.6. ABR Threshold Testing

The hearing condition of mice was detected by auditory brain stem response (ABR) thresholds. The mice used for ABR testing were fully anesthetized by a mixture of ketamine and xylazine. The initial dose of ketamine and xylazine was 85 and 15 mg/kg, respectively. The stimulus generation and biosignal acquisition parameters were similar to those used in a previous study [13]. Auditory stimuli were presented from a speaker 10 cm ahead of the mouse's head, needle electrodes were positioned in the vertex of the head and the reference, and grounding electrodes were positioned posterior to the external auditory canals. The tone bursts were generated by TDT RP2.1 Real-time signal processor, (10-ms duration with 0.5-ms rise/fall time presented at 21.1/s intervals) at 4, 8, 16, 24, and 32 kHz with SigGen software. The biological signals picked up by the electrodes were led to a RA4PA preamplifier from Tucker-Davis Technologies (TDT System III; Alachua, FL, USA). The evoked ABR responses were amplified 20x by a PA4 preamplifier (TDT) and averaged 1000 times. The intensity of tone bursts started at 90 dB SPL and declined by a step of 10 dB, except around threshold where it was 5 dB. The threshold was determined based on visibility and reproducibility of negative–positive waves. Threshold was defined as the lowest sound level that produces a reproducible response.

### 2.7. Statistical Analysis

The results are represented as the mean ± s.e.m., and statistical significance between groups was determined using an unpaired *t*-test or the Mann–Whitney *U* test. The GraphPad Prism software 8.0 was used for all analyses, and a ^∗^*p* < 0.05 was considered statistically significant.

## 3. Results

### 3.1. Dramatic Morphological Changes in the Stria Vascularis from *Slc26a4^−/−^* Mice

**Figure 1:**
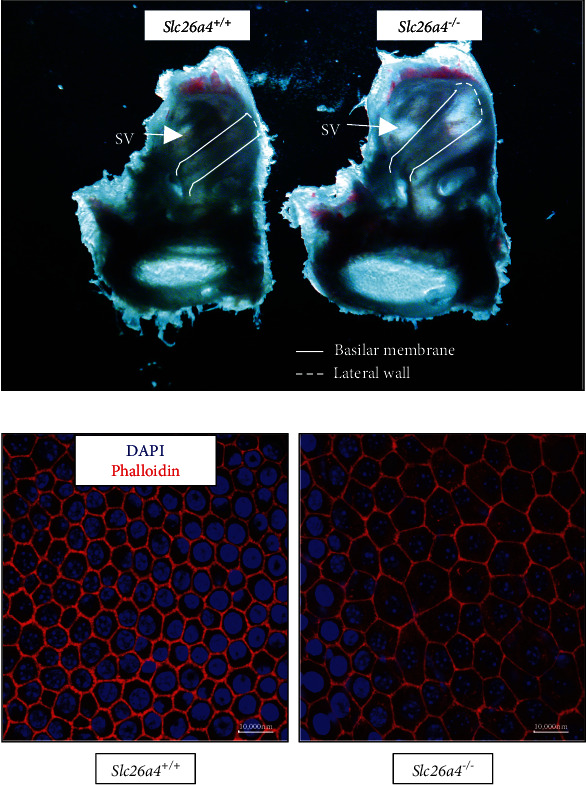
The dramatic morphological changes occurred in stria vascularis from *Slc26a4^−/−^* mice. (a) The scala media, formed by basilar membrane, lateral wall, and vestibular membrane, is significantly enlarged in *Slc26a4^−/−^* mice. SV: stria vascularis. The outline of stria vascularis, which is visualized through pigmentation in intermediate cells, is clearer in *Slc26a4^+/+^* mice. (b) Marginal cells in stria vascularis from wildtype or *Slc26a4^−/−^* mice were coated on coverlips and sent for phalloidin staining.

*Slc26a4* ^−/−^ mice typically have larger inner ears due to the dysfunction of the endolymphatic sac. Compared to those from *Slc26a4*^+/+^ mice, the cochlear lumen of the inner ears, isolated from 6-week-old *Slc26a4*^−/−^ mice, is significantly enlarged. Furthermore, the SV can be visualized through the pigmentation of the intermediate cells [14], and the lateral wall appears darker and more obvious in *Slc26a4*^+/+^ mice when compared to *Slc26a4*^−/−^ (Figure 1(a)).

The inner layer of the SV is formed by marginal cells facing the endolymph [10]. Therefore, we used the actin stain phalloidin, to profile the structure of these marginal cells in SV. Indeed, enlargement of marginal cells was seen in *Slc26a4*^−/−^ mice, while the marginal cells from *Slc26a4*^+/+^ mice were normal in both size and organization [15] (Figure 1(b)).

### 3.2. Transcript Regulation in the Stria Vascularis Caused by *Slc26a4* Deletion

**Figure 2:**
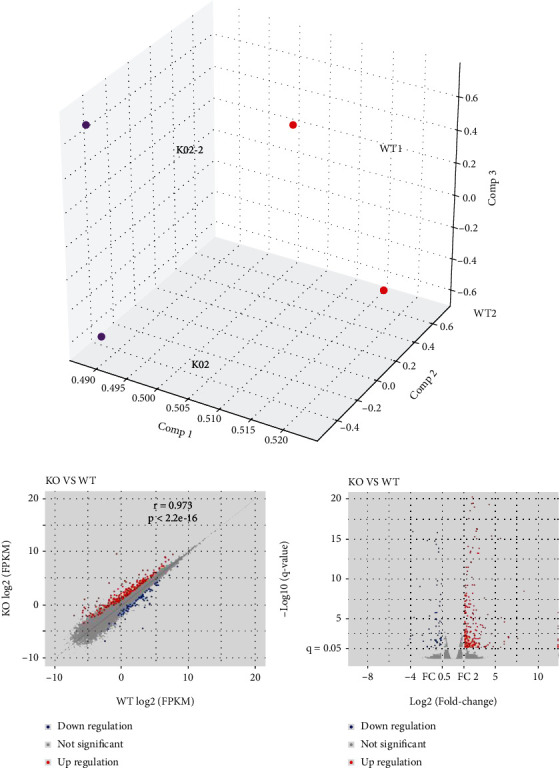
Transcripts regulated in stria vascularis of *Slc26a4* deletion. (a) Principal component analysis of RNAs from stria vascularis of wildtype (WT) and *Slc26a4^−/−^* (KO) mice. (b, c) Scatter plot (b) and Volcano plot (c) indicate individual RNAs sequenced.

To determine the role of *SLC26A4* in the function of the cochlea, wildtype and *Slc26a4^−/−^* mice were sacrificed and their SV were carefully isolated. Total RNA from pairs of SVs was extracted and then sent for next-generation sequencing and data from each sample generated on average 24-Mb of clean reads, after low-quality filtering, and these clean reads were mapped for reference. Each of the data sets contained 24-Mb reads and a mapping rate of 92–93%. Moreover, we counted the number of identified expressed genes and calculated their proportion and distribution to the total gene number in the database for each sample. The correlation for gene expression levels among the samples is a key criterion to determine whether the experiments are reliable and whether the samples chosen are reasonable, and principal component analysis was performed to assess gene expression levels (Figure 2(a)).

Volcano and Scatter plots showed differentially expressed transcripts for a fold change of >2 in the *Slc26a4* deficient group when compared to the wildtype group (183 upregulated genes and 63 downregulated genes) (Figures 2(b) and 2(c)).

### 3.3. Gene Ontology Analysis of the Differential Genes

**Figure 3:**
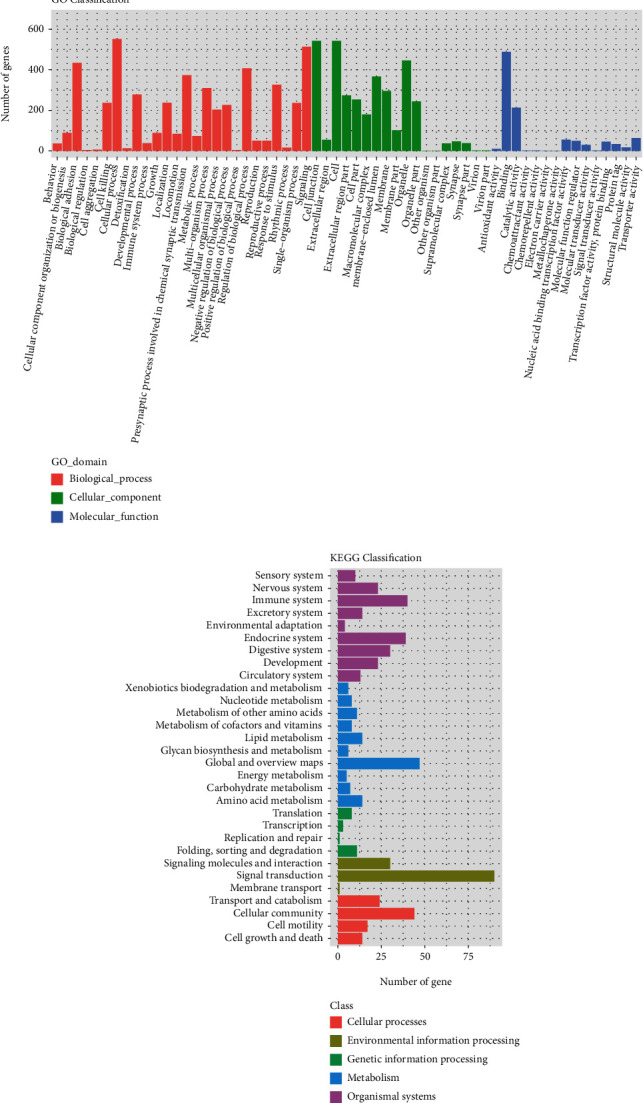
Gene ontology analysis and KEGG analysis of differentiated expressed genes in stria vascularis of *Slc26a4* deletion.

To better understand the associated functions of these differentially expressed genes in *Slc26a4*-mediated cochlear function, gene ontology (GO) analysis was used to perform enrichment analysis and classifications (Figures 3(a) and 3(b)). GO analysis identified enriched biological processes associated with “biological adhesion,” “cellular component organization or biogenesis,” “multicellular organismal processes,” and “developmental processes,” indicating that a strong multicellular organism biogenesis process occurred during the *Slc26a4*-mediated maintenance of cochlear homeostasis. Furthermore, enriched cellular component terms associated with “membrane,” “organelle,” “extracellular region,” and “membrane-enclosed lumen” were identified implying that diverse cellular components were involved in *Slc26a4*-mediated cochlear function. Enriched molecular functions were defined as associated with “structural molecule activity,” “catalytic activity,” and “molecular transducer activity,” indicating that formation of organelle structure and intracellular signal transduction were the major molecular functions for *Slc26a4*.

### 3.4. Analysis of Important KEGG Pathways

We next used the differential genes for KEGG pathway enrichment, and the results showed that genes involving in the “ECM-receptor interaction pathway,” “focal adhesion pathway,” “aldosterone-regulated sodium reabsorption pathways,” and “taste transduction pathways” were significantly enriched, indicating that loss of *Slc26a4* genes may lead to disrupted transmembrane cell communication and transport in the SV (Figures 3(c) and 3(d)).

Collectively, GO and KEGG pathway analysis suggested an essential role for *SLC26A4* in the regulation of extracellular structure formation, cell-to-cell or cell-to-ECM adhesion, and transmembrane transport.

### 3.5. Validation of Selected Genes

**Figure 4:**
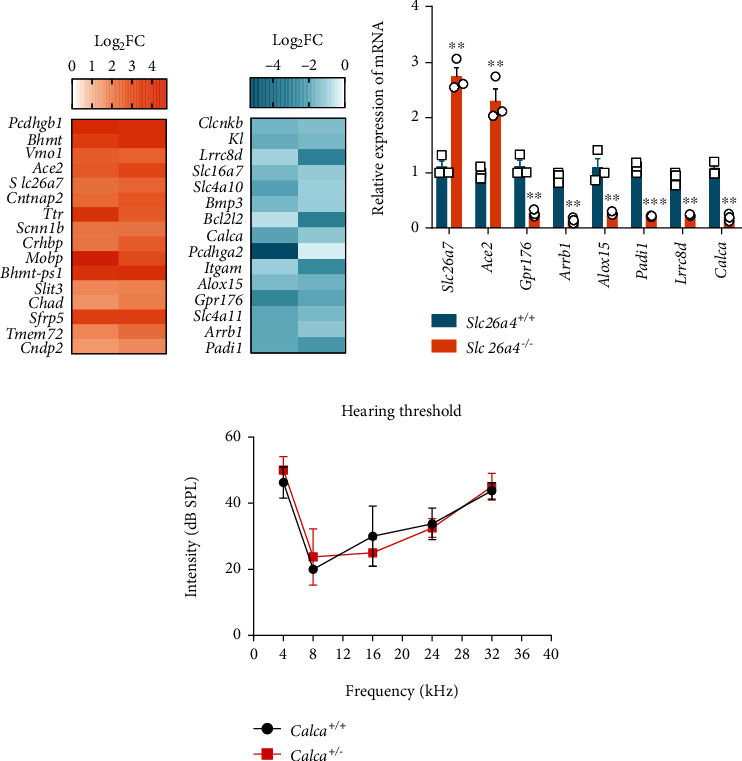
Validation of differentiated expressed genes in stria vascularis of *Slc26a4* deletion. (a, b) Heatmap indicated selected upregulated genes (a) and downregulated genes (b) in *Slc26a4^−/−^* SVs. (c) Quantitative RT-PCR analysis of expression of indicated genes in *Slc26a4^−/−^* SVs. (d) ABR test of wildtype and *Calca^+/-^* mice. ^∗^*p* < 0.05 and ^∗∗^*p* < 0.01 by the unpaired *t*-test (c). Data are from three independent experiments with biological duplicates in each (c, d; mean ± s.e.m. of *n* = 3 duplicates).

Several important upregulated and downregulated genes are represented as shown in the heatmap (Figures 4(a) and 4(b)). Quantitative RT-PCR results validated the differential expression of selected candidate genes, including *Padi1*, *Arrb1*, *Ace2*, *Gpr176*, *Lrrc8d*, *Alox15*, and *Calca* (Figure 4(c)).

We noted that one of the important downregulated genes in the SV of *Slc26a4* deficient mice was *Gpr176*, which encodes a G-protein-coupled receptor [16] and has previously been reported to be associated with adult-onset autosomal dominant cerebellar ataxia, with deafness and narcolepsy [17]. Quantitative RT-PCR analysis confirmed the downregulation of *Gpr176* in the SV of *Slc26a4^−/−^* mice (Figure 4(c)), suggesting a reduction of *GPR176*-mediated signaling upon *Slc26a4* deficiency.

Another important gene found was *Calca* and therefore, we generated a *Calca* knockout mouse using CRISPR/Cas9 technology and carefully examine its ABR threshold. The results showed that *Calca^+/-^* mice had equivalent hearing to the wildtype controls (Figure 4(d)), suggesting that at least heterozygous deletion of *Calca* had no significant effect on hearing.

### 3.6. Altered Hcy Metabolism in the Stria Vascularis of *Slc26a4^−/−^* Mice

**Figure 5:**
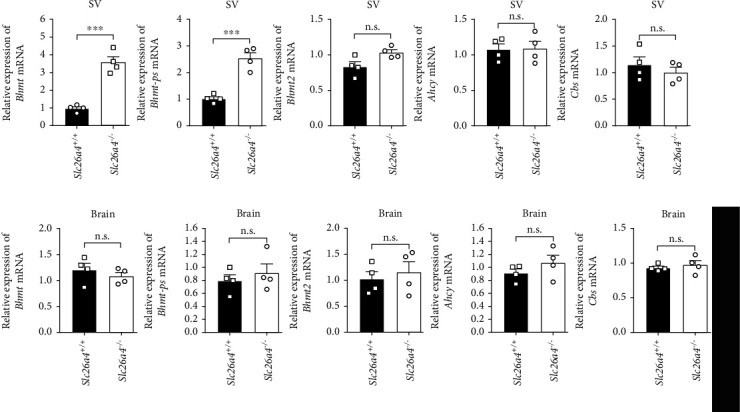
Altered expression of Hcy metabolism enzymes in *Slc26a4^−/−^* SVs. (a, b) Quantitative RT-PCR analysis of expression of Hcy metabolism enzymes in *Slc26a4^−/−^* SVs (a) or brains (b). n.s.: no significance. ^∗^*p* < 0.05 and ^∗∗^*p* < 0.01 by the unpaired *t*-test (c). Data are from three independent experiments with biological duplicates in each (a, b; mean ± s.e.m. of *n* = 3 duplicates).

**Figure 6:**
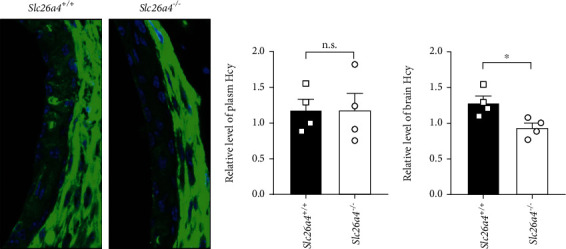
Altered Hcy metabolism in *Slc26a4^−/−^* SVs. (a) Stria vascularis from wildtype or *Slc26a4^−/−^* mice were coated on coverlips and sent for immunostaining against Hcy. (b, c) Elisa assay of Hcy level in plasma (b) and brain (c) from wildtype or *Slc26a4^−/−^* mice. n.s.: no significance. ^∗^*p* < 0.05 by the unpaired *t*-test (b, c). Data are from three independent experiments with biological duplicates in each (a, b, c; mean ± s.e.m. of *n* = 3 duplicates).

We next determined the expression of *Bhmt*, as well as several other enzymes required for Hcy metabolism including *Bhmt2*, *Ahcy*, and *Cbs* in the SV and brain from *Slc26a4^−/−^* mice using quantitative RT-PCR. The results showed that *Slc26a4* deficiency significantly increased *Bhmt* expression in the SV but not the brain, while the expressions of other enzymes involved in Hcy metabolism were similar in both groups (Figures 5(a) and 5(b)). Interestingly, we also observed a significant increase in the *Bhmt* pseudogene (*Bhmt-ps)*, in the SV of *Slc26a4^−/−^* mice but not in brain, which was similar to the levels of *Bhmt* itself, suggesting that there may be a common regulation of *Bhmt* and *Bhmt-ps* in the SV. Thus, we applied immunostaining assays using an anti-Hcy polyclonal antibody to measure levels in the SV from wildtype and *Slc26a4^−/−^* mice and found that Hcy levels were dramatically decreased in the SV of *Slc26a4^−/−^* mice (Figure 6(a)). However, we did not observe a significant change in serum Hcy levels in these mice when compared to the wild types, based on an anti-Hcy ELISA (Figures 6(b) and 6(c)).

## 4. Discussion

Hearing loss could be caused by genetic factors, aging, chronic cochlear infections, infectious diseases, ototoxic drugs, and noise exposure [18–24]. Most of the hearing loss is due to the damage of hair cells and spiral ganglion neurons and has been extensively investigated in many previous studies [25–30], while the detailed mechanism of stria vascularis-related hearing loss, such as Pendred syndrome, has not been well known. *SLC26A4* affects cochlear function both in humans and mice; however, the mechanism by which *SLC26A4* achieves this is unclear [15]. Transcriptome sequencing has been extensively used in many previous studies to investigate the detailed mechanism in the inner ear [31–37]. In our study, we applied a transcriptome sequencing approach to examine the levels of differentially expressed genes in the SV from *Slc26a4^−/−^* mice. Using bioinformatic analysis, we identified 183 upregulated genes and 63 downregulated genes in the SV associated with *Slc26a4* depletion. Furthermore, the cellular functions of these genes are related to cell communication, extracellular matrix organization, and transmembrane transportation. Therefore, our findings suggest a novel mechanism by which *SLC26A4* can affect cochlear function.

Previous studies have shown that macrophage invasion contributes to degeneration of the SV in the *Slc26a4^−/−^* mouse model [14, 38]. Consistent with this, we found an additional two well-characterized inflammation regulating genes, *Alox15*, that encoded arachidonate 15-lipoxygenase [39] and *Arrb1*, encoding *β*-arrestin 1 [40, 41], in top downregulated genes in the SV of *Slc26a4^−/−^* mice. Quantitative RT-PCR results also showed that the expression levels of *Arrb1* and *Alox15* were decreased in the SV of these knockout mice. These findings suggest a possible role for tissue inflammation in the degeneration of the SV in the Pendred syndrome mouse model [42, 43].

The *Calca* gene, encoding calcitonin and alpha-calcitonin gene-related peptide [44], is one of the top downregulated genes found in our study. As reported in a previous study, *Calca* is expressed in mouse cochlea at an early stage during development [45]. We therefore obtained a *Calca* mutant mouse to determine whether the low expression of *Calca* affected hearing. Since *Calca* plays a critical role in multiorgan development [46, 47], *Calca^−/−^* mice, created by CRISPR/Cas9 methodology, were unhealthy and died soon after birth. Therefore, we were forced to generate a *Calca*^+/-^ heterozygous mouse model to determine hearing. The ABR result showed that there was no difference in the hearing threshold between the wildtype and the *Calca*^+/-^ animals. No significant hearing loss may be due to the heterozygotic nature of the mice used. Therefore, further research is needed to determine whether more subtle, or hidden hearing loss is present in these mice. Also, a new *Calca* minus model is needed to further investigate its effect upon hearing.

Our transcriptome sequencing results found that the most distinctive upregulated gene in the SV from *Slc26a4^−/−^* mice was *Bhmt*, which encodes betaine Hcy S methyltransferase, whose activity is required for the transfer of a methyl group from betaine to Hcy, a nonproteinaceous sulfur amino acid [48, 49]. Malfunction of *Bhmt* leads to hyperhomocysteinemia and several previous epidemiological and experimental studies have revealed a correlation between hyperhomocysteinemia and hearing loss [50]. Previous study showed that Hcy level plays an important part in keeping the integrity of SV, which is the foundation of SV to establish the EP [51]. We consistently observed a dramatic decrease in Hcy from the SV of *Slc26a4^−/−^* mice when compared to wildtypes, and recently, nutritional imbalance is emerging as a causative factor associated with hearing loss. Furthermore, Teresa Partearroyo et al. have reported that *Bhmt* plays a central role in the homeostasis of methionine metabolism in the cochlea and its deficiency in mice causes increased susceptibility to noise-induced hearing loss [52, 53]. Moreover, a decrease in *Bhmt* expression and an increase of Hcy in the SV is thought to explain the hearing loss phenotype seen in *Cx30^−/−^* mice [54]. However, one difference between our findings and previous studies is that in the SV of *Slc26a4^−/−^* mice, *Bhmt* is upregulated, which leads to decreased amounts of Hcy, which is part of the folate and methionine cycles. The elevated expression of *Bhmt* leads to an increased consumption of Hcy and folate and production of methionine and S-Adenosylmethionine, which serve as the major donors of methyl groups in the synthesis of hormones, nucleotides, and membrane lipids. It is speculated that the altered expression of *Bhmt* results in a dramatic change in multiple biochemical reactions and a disruption of nutrient homeostasis in the endolymph. However, whether the decrease in Hcy levels accounts for cochlear dysfunction and hearing loss in Pendred syndrome mouse models requires further investigation.

## 5. Conclusion

Transcriptomic profiling revealed that *Slc26a4* deficiency significantly affected the expression of genes associated with cell adhesion, transmembrane transport, and the biogenesis of multicellular organisms. The altered expression of *Bhmt* results in a dramatic change in multiple biochemical reactions and a disruption of nutrient homeostasis in the endolymph which may contribute to hearing loss of *Slc26a4* knockout mouse.

## Figures and Tables

**Table 1:** Primer used in this study.

Target	Forward (5'to3')	Reversed (5'to 3')
*Slc26a7*	GCATGATGAAACCTCGCAACA	TTCATTGCAGTTGCCGTTGG
*Ace2*	CACTCCTGCCACACCACGTT	TGGTCTTTAGGTCAAGTTTACAGCC
*Gpr176*	GGTGTTATGGTCAACTTGCCG	AGAGCATCGTATAGATCCACCAG
*Lrrc8d*	ATGTTTACCCTTGCGGAAGTTG	CATCAGCATAACGACTGCCAG
*Padi1*	TGTGTGCGTGGTAGGTGTG	TCGAGGGATCGTAGACCATGT
*Arrb1*	AAGGGACACGAGTGTTCAAGA	CCCGCTTTCCCAGGTAGAC
*Alox15*	CAGGGATCGGAGTACACGTT	GATTGTGCCATCCTTCCAGT
*Bmht*	CTGGGGAAGTGGTTTGGACA	GCCGGAAGCTATTCGCAGAT
*Bhmt2*	CTCCAGAAGCAGTGGTAGAACATC	CATCAGCTCCCGCTCTCAAG
*Ahcy*	TCGAAGTGTCCAATGTTACAGAC	CTTGGCCGGCACTTTGAG
*Cbs*	GCAGCGCTGTGTGGTCATC	GTCACTCAGGAACTTGGACATGTAGT

## Data Availability

Data are available from the author Wenyue Xue (xuecindy0107@163.com) upon reasonable request and with permission of the Department of Otolaryngology-Head and Neck Surgery, Shanghai Jiao Tong University Affiliated Sixth People's Hospital.
